# Consumer-Grade Wearable Device for Predicting Frailty in Canadian Home Care Service Clients: Prospective Observational Proof-of-Concept Study

**DOI:** 10.2196/19732

**Published:** 2020-09-03

**Authors:** Ben Kim, Sandra M McKay, Joon Lee

**Affiliations:** 1 School of Public Health and Health Systems University of Waterloo Waterloo, ON Canada; 2 VHA Home Healthcare Toronto, ON Canada; 3 School of Physical Therapy University of Toronto Toronto, ON Canada; 4 Data Intelligence for Health Lab Cumming School of Medicine University of Calgary Calgary, AB Canada; 5 Department of Community Health Sciences Cumming School of Medicine University of Calgary Calgary, AB Canada; 6 Department of Cardiac Sciences Cumming School of Medicine University of Calgary Calgary, AB Canada

**Keywords:** frailty, mobile health, wearables, physical activity, home care, prediction, predictive modeling, older adults, activities of daily living, sleep

## Abstract

**Background:**

Frailty has detrimental health impacts on older home care clients and is associated with increased hospitalization and long-term care admission. The prevalence of frailty among home care clients is poorly understood and ranges from 4.0% to 59.1%. Although frailty screening tools exist, their inconsistent use in practice calls for more innovative and easier-to-use tools. Owing to increases in the capacity of wearable devices, as well as in technology literacy and adoption in Canadian older adults, wearable devices are emerging as a viable tool to assess frailty in this population.

**Objective:**

The objective of this study was to prove that using a wearable device for assessing frailty in older home care clients could be possible.

**Methods:**

From June 2018 to September 2019, we recruited home care clients aged 55 years and older to be monitored over a minimum of 8 days using a wearable device. Detailed sociodemographic information and patient assessments including degree of comorbidity and activities of daily living were collected. Frailty was measured using the Fried Frailty Index. Data collected from the wearable device were used to derive variables including daily step count, total sleep time, deep sleep time, light sleep time, awake time, sleep quality, heart rate, and heart rate standard deviation. Using both wearable and conventional assessment data, multiple logistic regression models were fitted via a sequential stepwise feature selection to predict frailty.

**Results:**

A total of 37 older home care clients completed the study. The mean age was 82.27 (SD 10.84) years, and 76% (28/37) were female; 13 participants were frail, significantly older (*P*<.01), utilized more home care service (*P*=.01), walked less (*P*=.04), slept longer (*P*=.01), and had longer deep sleep time (*P*<.01). Total sleep time (r=0.41, *P*=.01) and deep sleep time (r=0.53, *P*<.01) were moderately correlated with frailty. The logistic regression model fitted with deep sleep time, step count, age, and education level yielded the best predictive performance with an area under the receiver operating characteristics curve value of 0.90 (Hosmer-Lemeshow *P*=.88).

**Conclusions:**

We proved that a wearable device could be used to assess frailty for older home care clients. Wearable data complemented the existing assessments and enhanced predictive power. Wearable technology can be used to identify vulnerable older adults who may benefit from additional home care services.

## Introduction

Frailty has detrimental health impacts among community-dwelling older adults. Frailty is associated with higher mortality [1-3], functional impairment [4,5], hospitalization [2,3], long-term care facility admission [3], and disability in activities of daily living [4]. Frailty also increases the demand on formal and informal caregivers, including home and community care services and family members [6]. A recent study [7] identified that caregiver burden can be predicted based on the physical frailty level of geriatric patients. Due to its significant impact on health outcomes and its burden on health care systems, improved screening and monitoring of frailty for community-dwelling older adults is deemed vital [8].

The prevalence of frailty among community-dwelling older adults is poorly understood. A systematic review [9] reported that its prevalence ranges between 4.0% and 59.1%; varying operational definitions and the heterogeneity of tools used in the studies resulted in a wide range of estimates. However, the prevalence range narrows to 4.0% to 17.0% when only the prevalence of physical phenotype frailty is aggregated by excluding social or cognitive deficits [9].

Both home and community health care are challenged with increased demand, primarily due to the aging population and emphasis on aging-in-place [10]. The demand for home and community health care service is expected to continue to rise in an effort to keep patients in their own community to reduce health care costs [11]. Screening and monitoring frailty in this population can benefit the home and community health care sector in multiple ways. Effective frailty intervention programs involve lifestyle changes including improving nutrition, increasing physical activity, and modifying the home environment [12]. Home and community health care clinicians are uniquely situated to deliver and monitor such interventions in a longitudinal manner, which can contribute to successful lifestyle changes. Screening for frailty at the community level can also help the home and community health care sector to identify vulnerable groups and allocate resources more efficiently [13].

Tools to screen community-dwelling older adults for frailty exist, but they have been used inconsistently and are often impractical or have been invalidated [14]. Wearable devices have been suggested as a potential tool to monitor frailty, and a few research studies [15-18] have explored this possibility. These studies explored the feasibility of using research-grade wearable devices, such as ActiGraph or independently developed wearable devices. These studies provide evidence for the internal construct validity of research-grade wearable devices to screen for frailty [19], as well as for a strong association between varying sleep quality parameters and frailty [20-22]. Consumer-grade wearable devices are a promising tool to monitor frailty as they have become smaller, cheaper, and ever more accessible in recent years [23], with older adults being the fastest growing group of wearable device users [24]. Research studies [25-27] have demonstrated the reliability of these devices for measuring step count, sleep quality, and heart rate compared to gold standard measures that are used in laboratory and clinical settings. Further validation studies demonstrated a high agreement between consumer-grade and medical-grade devices among specific populations, including patients with chronic obstructive pulmonary disease [28], pediatric patients [29], patients in intensive care units [30], and patients in cardiac rehabilitation [31].

Recognizing the need for an innovative solution to measure frailty in community-dwelling older adults, we set out to investigate the possibility of using consumer-grade wearable devices. We examined the data generated from a wearable device worn by home care clients to identify associations with frailty. We also aimed to identify key wearable device measures that can predict the status of frailty. Study procedure, tools, and statistical analyses are described. The results of the study are then presented, followed by a discussion where new findings are interpreted and compared to existing knowledge. The implications for frailty research studies, for wearable device research studies, and in home and community health care sectors, as well as the limitations of the study are presented.

## Methods

### Study Design

A prospective observational study was conducted to meet the study objectives. Participants were asked to wear a wearable device for a minimum of 8 days. At the end of the study, participants were assessed for frailty, activities of daily living, and level of comorbidity.

### Recruitment

Home care clients in the Greater Toronto Area were recruited through VHA Home Healthcare from August 2018 to September 2019. VHA Home Healthcare is a home care agency that serves over 3000 clients throughout the Greater Toronto Area and other metropolitan areas in Ontario, Canada. Patients 55 years or older who had been receiving personal support service for more than 3 months were eligible for the study. Patients who were diagnosed with primary neuromuscular pathology, dependent on wheelchair, in an end-of-life program, or had cognitive impairments that could interfere with the use of wearable devices were excluded. Eligible home care patients were identified using VHA’s electronic medical record system.

### Wearable Device

The Xiaomi Mi Band Pulse 1S (Mi Band, hereafter) is a commercially available wearable device that is worn on the wrist. It uses a triaxial accelerometer to capture motions to approximate step count and sleep events. It is equipped with an optical heart rate sensor (photoplethysmography) to measure minute-by-minute heart rate. While the Mi Band can be worn on either the wrist or neck (as a pendant), its placement was limited to the wrist for the study. The reliability and internal consistency of Mi Band’s performance for measuring step count when walking and jogging has been validated [32,33]. Wrist-worn wearable devices displayed systematically lower heart rate during exercise, but the Mi Band demonstrated the highest accuracy [32].

We collected daily step count, light sleep time, deep sleep time, total sleep time, awake time, sleep quality, mean heart rate, and heart rate standard deviation. Sleep quality was calculated as the percentage of sleep duration over total sleep time; sleep duration was determined by subtracting awake time from total sleep time [34,35]. Heart rate was measured in beats per minute. A pool of 10 devices were used in rotation and sanitized throughout the study. The adherence to wearing the device was defined as 10 hours or more of wear time per day [36].

### Frailty Assessment

Frailty was assessed using the Fried Frailty Index, a tool that has been developed for and used widely with community-dwelling older adults [1]. The Fried Frailty Index assesses phenotypic frailty based on 5 criteria: weight loss, exhaustion, slowness, weakness, and low physical activity. The index categorizes frailty into 3 stages based on the number of criteria that are met: nonfrail, prefrail, and frail corresponding to scores of 0, 1-2, and 3-5, respectively [1]. We dichotomized the Fried Frailty Index into a frail group for those with a score of 3 or higher and a nonfrail group for those with a score of 2 or lower [1].

### Other Variables

Sociodemographic variables were collected using a short background questionnaire and through review of the patient’s medical chart. These sociodemographic variables included age, sex, weight, height, ethnicity, level of education, income, and marital status. The level of comorbidity was assessed using the Charlson Comorbidity Index (CCI) [37]. The level of activities of daily living was assessed with the Katz index of independence [38]. The number of hours of service received per week was collected by reviewing the patient’s medical chart.

### Statistical Analysis

Descriptive statistics and univariate comparisons of means, medians, and proportions were performed to describe the sociodemographic information and patient assessments according to their frailty status. The level of education was condensed into 2 levels: high school (some or completed) and postsecondary. Household income was categorized into a lower income, those who earned $30,000 (approximately US $22,653) per year or less, and higher income, those who earned $30,000 or higher per year. Ethnicity was categorized into 2 levels: Caucasian and others which included aboriginal identity, Latin American, African American, South Asian, Southeast Asian, East Asian, Filipino, Arab, and West Asian.

Wearable device data were examined for participants adherence level, and days with less than 10 hours of wear time were excluded. Heart rate measurements of zero were generated when the device failed to have good skin contact. Such measurements were treated as missing and were removed from the analyses.

The Shapiro-Wilk test was performed to check for normality. To check for significant differences between patients who were frail and patients who were nonfrail, when the assumption of normal distribution was met, a two-tailed independent *t* test was used, while the Mann-Whitney *U* test was performed otherwise. The chi-square test was performed for categorical variables. The posthoc chi-square test was performed when significance was observed.

Pearson and Spearman correlation statistics were used to examine the relationship between frailty, sociodemographic information, patient assessments, and the data collected from the wearable devices.

Multiple logistic regression models were generated to predict frailty status. A sequential stepwise feature selection method was used to select the variables to be fitted into the models. The feature selection was used on the pool of sociodemographic and patient assessment variables to determine the features to be included in model 1. Model 2 was built by applying feature selection to the variables derived from the wearable device data. Model 3 used all available variables in a feature selection algorithm; the selected variables were used to build the logistic regression model. The Hosmer-Lemeshow test was performed to test the goodness-of-fit for each model. The predictive performance of each model was evaluated and compared using the area under the receiver operating characteristics curve (AUROC).

Statistical significance was set at α=.05 for all statistical results. The significance level for posthoc tests was corrected using the Bonferroni method. All statistical analyses were performed using R (version 3.6.0) in R studio (version 1.2.1335; R Studio Inc). Stepwise feature selection was performed using the function (stepAIC, version 7.3-51.4) from the MASS library [39].

### Ethics, Consent, and Permissions

This study received ethics approval from the Office of Research Ethics Board at the University of Waterloo (ORE22842).

## Results

### Recruitment

A total of 72 older adults responded to the mailed recruitment brochure. All 72 older adults were contacted, and 45 agreed to participate in the study; 4 participants withdrew before completion of the 8-day study period. Data attrition due to technical issues resulted in data from 4 participants not being included. In total, 37 older home care clients were included in the study.

### Participant Characteristics

Participants were 57 to 96 years of age, with a mean age of 82.23 (SD 10.84) years and 76% (28/37) were female (Table 1). The prevalence of frailty among the study population was 35% (13/37). On average, participants were observed for 9.43 (SD 1.99) days. Participants who were frail (mean age: 83.92 years) were significantly older (*P*<.001) than those who were nonfrail (mean age: 80.61 years). There was a significant difference in the income level between older adults who were frail and those who were nonfrail (*P*=.03). Posthoc comparisons within each of the 3 income levels showed no statistical significance (low income: *P*=.93; mid to high income: *P*>.999) after correcting the α level with the Bonferroni method. Frail patients received significantly greater hours of home care services per week compared to the hours received by patients who were nonfrail (*P*=.01). The resulting *P* values of the Shapiro-Wilk normality tests are presented in Multimedia Appendix 1. The results of group difference tests are presented in Multimedia Appendix 2.

**Table 1:** Baseline sociodemographic and patient characteristics stratified by frailty status.

Characteristics		Frail (n=13)	Nonfrail (n=24)	*P* value
Age (years), mean (SD)		83.92 (9.66)	80.61 (13.96)	<.001^a^
**Sex, n (%)**				>.999^b^
	Male	3 (23)	6 (25)	
	Female	10 (77)	18 (75)	
BMI (kg/m^2^), mean (SD)		26.96 (6.70)	28.54 (5.43)	.44^c^
ADL^d^ score, mean (SD)		4.62 (1.45)	5.08 (0.88)	.43^a^
CCI^e^ score, mean (SD)		1.92 (1.26)	1.25 (1.11)	.11^a^
**Marital status, n (%)**				.29^b^
	Single	1 (8)	7 (29)	
	Divorced or separated	2 (15)	5 (21)	
	Widowed	4 (31)	7 (29)	
	Currently married	6 (46)	5 (21)	
**Education, n (%)**				.12^b^
	High school or less	8 (62)	7 (29)	
	Postsecondary or higher	5 (38)	17 (71)	
**Income, n (%)**				.03^b^
	Prefer not to answer	7 (54)	3 (12)	.06^f^
	Low income	4 (31)	13 (54)	.93^f^
	Mid to high income	2 (15)	8 (33)	>.999^f^
**Ethnicity, n (%)**				.71^b^
	White	10 (77)	21 (88)	
	Other	3 (23)	3 (12)	
Personal support service, hours per week		5.15 (3.51)	2.77 (1.85)	.01^a^

^a^Mann-Whitney *U* test was used.

^b^Chi-square test was used.

^c^An independent *t* test was used.

^d^ADL: activities of daily living; Katz index of independence was used.

^e^CCI: Charlson Comorbidity Index.

^f^Posthoc chi-square test was used.

### Frailty and Wearable Device Data

On average, older adults wore the device for 20.03 (1.64) hours per day (Table 2). Home care clients who were frail reported significantly lower daily step counts than their nonfrail counterparts did (mean steps per day: 367.11 vs. 1023.95, respectively; *P*=.04). Total sleep time (*P*=.01) and deep sleep time (*P*<.01) were significantly longer for older adults who were frail, but no difference was found for light sleep time (*P*=.04). No difference was found for heart rate measures. Box plots corresponding to Table 2 are presented in Multimedia Appendix 3.

**Table 2:** Difference in the data collected from the wearable device between frail and nonfrail participants.

Measures		Frail (n=13), mean (SD)	Nonfrail (n=24), mean (SD)	*P* value
Worn time (hours per day)		20.66 (1.03)	19.69 (1.82)	.16^a^
Daily step count		367.11 (272.63)	1023.95 (863.83)	.04^a^
**Sleep measures**				
	Deep sleep time (minutes)	138.90 (64.00)	75.65 (39.12)	<.001^a^
	Light sleep time (minutes)	350.88 (130.56)	312.78 (82.32)	.35^b^
	Total sleep time (minutes)	489.78 (139.54)	388.44 (93.28)	.01^a^
	Awake time (minutes)	36.03 (24.27)	65.05 (57.97)	.17^a^
	Sleep quality (%)	92.48 (5.62)	78.95 (26.53)	.08^a^
**Heart rate measures**				
	Heart rate (bpm)	82.77 (10.25)	77.43 (8.66)	.13^b^
	Heart rate SD (bpm)	22.12 (7.61)	18.78 (4.54)	.17^b^

^a^Mann-Whitney *U* test was used.

^b^An independent *t* test was used.

### Factors Correlated With Frailty

The correlation between wearable data and frailty is summarized in Table 3. Daily step count was negatively correlated with frailty level (*r*=–0.52, *P*<.001). All 5 sleep measures were moderately correlated with frailty. Education level was moderately correlated with frailty status (*r*=–0.40, *P*=.02). No relationship was found between heart rate measures and frailty status.

**Table 3:** Correlations between wearable device data, patient characteristics, and frailty.

		Frailty	
		Correlation coefficient	*P* value
Daily step count		–0.52	.001
**Sleep measures**			
	Total sleep time	0.52	.001
	Deep sleep time	0.47	.003
	Light sleep time	0.35	.03
	Sleep quality	0.56	<.001
	Awake time	–0.54	<.001
**Heart rate measures**			
	Mean heart rate	0.11	.54
	Heart rate SD	–0.25	.16
**Sociodemographic**			
	Age	0.29	.08
	Sex	0.074	.66
	BMI	–0.068	.69
	Income level	–0.066	.74
	Education level	–0.40	.02
**Patient assessments**			
	ADL^a^ score	–0.18	.27
	CCI^b^ score	0.16	.33
Personal support hours		0.23	.17

^a^ADL: activities of daily living; Katz index of independence was used.

^b^CCI: Charlson Comorbidity Index.

### Frailty Prediction

#### Model Description

A total of 3 multiple variable logistic regression models were fitted to predict frailty with the sociodemographic variables, patient assessments, and wearable data (Table 4). Income was excluded from the feature selection method since a high number of participants declined to answer. Model 1 formulation began by fitting the sociodemographic variables and patient assessments. The feature selection method resulted in a model that contains CCI and education level. Model 2 used variables derived from the wearable device data only. The resulting model was fitted with step count, deep sleep time, awake time, and heart rate standard deviation. Model 3 used all available variables and was fitted with deep sleep time, step count, age, and education level.

**Table 4:** Three frailty prediction models and the variables selected by the stepwise feature selection method.

Models	Variable pool	Selected variables
Model 1	Sociodemographic and patient assessment variables	CCI^a^, education level
Model 2	Wearable device–derived variables	Step count, deep sleep time, light sleep time, heart rate standard deviation
Model 3	Sociodemographic, patient assessment, and wearable device–derived variables	Deep sleep time, step count, age, education level

^a^CCI: Charlson Comorbidity Index.

#### Model Evaluation

Table 5 shows the results of multiple logistic regression analyses and the factors predictive of frailty. Model 1 showed no significant association. For model 2, deep sleep time was a significant predictor of frailty (*P*<.01). Increasing deep sleep time was significantly associated with increased odds of frailty (adjusted odds ratio [OR] 1.02, 95% CI 1.01-1.05, *P*<.01). For model 3, deep sleep time (*P*=.02) and age (*P*=.03) were significant predictors. Increasing deep sleep time was associated with an increase in the odds of frailty (adjusted OR 1.03, 95% CI 1.01-1.07, *P*=.02), whereas increasing age was associated with a decrease in the odds of frailty (adjusted OR 0.90, 95% CI 0.80-0.99, *P*=.03).

All 3 models were evaluated for their goodness of fit using the Hosmer-Lemeshow statistic. Overall, no model showed statistical significance on this test, indicating they had acceptable goodness-of-fit, and the predicted frailty matched the observed frailty status (Table 6).

**Figure 1:**
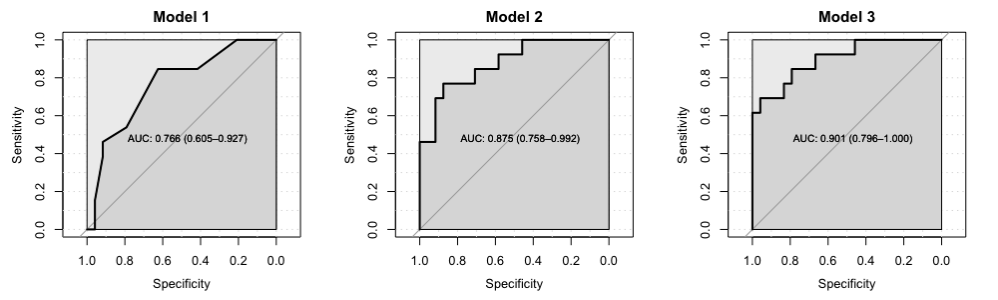
The receiver operating characteristics curves (with area under the curve) for all models fitted to predict frailty. AUC: area under the curve.

When the predictive performance was evaluated by AUROC, all 3 models showed medium to high values. Model 1 (AUROC 0.77), based on sociodemographic and patient assessment variables, was outperformed by model 2 (AUROC 0.88), which was fitted with wearable device variables. Model 3 (AUROC 0.90) had the best predictive performance (Table 6). The receiver operating characteristic curves are shown in Figure 1 for each model.

**Table 5:** Multiple logistic regression of factors associated with frailty.

Model and variables		Adjusted OR^a^ (95 % CI)		*P* value	
**Model 1**					
	CCI^b^		1.78 (0.95, 3.66)		.09
	Education level—high school or below		reference		—
	Education level—postsecondary education or higher		0.22 (0.04, 0.96)		.05
**Model 2**					
	Step count		1.00 (1.00, 1.00)		.17
	Deep sleep time		1.02 (1.01, 1.05)		.02
	Awake time		0.97 (0.93, 1.01)		.18
	Heart rate standard deviation		1.17 (0.99, 1.46)		.10
**Model 3**					
	Deep sleep time		1.03 (1.01, 1.07)		.04
	Step count		1.00 (1.00, 1.00)		.06
	Age		0.90 (0.80, 0.99)		.04
	Education level—high school or less		reference		—
	Education level—postsecondary education or higher		0.11 (0.01, 0.94)		.06

^a^OR: odds ratio.

^b^CCI: Charlson Comorbidity Index.

**Table 6:** Summary of model performance in predicting frailty status.

Models	Accuracy	Sensitivity	Specificity	AUROC^a^	Hosmer-Lemeshow test *P* value
Model 1: Sociodemographic and patient assessment variables	0.76	0.46	0.92	0.77	0.73
Model 2:Wearable device derived variables	0.81	0.69	0.88	0.88	0.95
Model 3: All variables from models 1 and 2	0.81	0.69	0.88	0.90	0.85

^a^AUROC: area under the receiver operating characteristics curve.

## Discussion

### Principal Findings

The growing aging population in Canada and the emphasis on aging-in-place call for innovative ways to improve efficiency in the home and community health care sector. There is an increasing interest in integrating information and communication technology such as consumer-grade wearable devices into health care delivery due to their rising popularity, ease-of-use, and the potential usefulness of continuously collected data [40]. The aim of this study was to investigate the possibility of assessing and predicting frailty using a wearable device.

We observed 37 older home care clients for a minimum of 8 days. The prevalence of frailty in the study sample, 35% (13/37), was similar to that found in other research studies examining home care clients [3,41]. Many research studies [9,42] reported a significantly higher prevalence of frailty in older women compared to prevalence in older men, but this was not observed in our study sample. However, another study [3] that examined the same population did not find any significant difference between the sexes. Overall, the study sample seemed reasonably representative of the home care population. Previous research studies [1,43] reported an association between income and education level and frailty. Our study sample had significantly different income levels between the 2 frailty groups (*P*=.03). However, the posthoc chi-square analysis results did not reach statistical significance (low income: *P*=.93; mid to high income: *P*>.999). Education level was moderately correlated with frailty level (r=–0.40, *P*=.02). Overall, our study sample displayed the general characteristics of frail populations [1,43].

Our study found a significantly higher utilization of home care service by older adults who were frail compared to utilization by older adults who were nonfrail (mean hours per week: 5.15 vs 2.77; *P*=.01). Unfortunately, the current system fails to meet all care needs of home care clients as indicated by the increased hours of informal care and caregiver distress for the home care clients with more severe frailty [13]. Resulting adverse health outcomes and increased health care utilization [3] highlight the need for a better allocation of home care service to those who stand to benefit the most.

In our study sample, older adults who were nonfrail walked significantly more than the older adults who were frail. This result is in line with the findings of previous research studies where reduced daily step count and physical activity were observed for frail community-dwelling older adults [44] and ICU patients [45]. In a previous study [46], daily step count was significantly related to frailty. Our study extended this evidence outside the controlled settings and beyond 24-hour monitoring period [47,48], and demonstrated the relationship in an unsupervised setting.

Sleep measures including longer total sleep, deep sleep, and light sleep durations; awake time; and sleep quality were shown to be related to more severe frailty. This is contrary to the common knowledge of deterioration of sleep quality and quantity with aging [49]. However, in epidemiological studies, a longer sleep duration was associated with an increased risk of heart disease and all-cause mortality [50]. The lowest mortality risk was found for those who sleep about 7 hours a night [51], while men who slept more than 8 hours per day had a tripled risk of heart disease [52]. This relationship was shown in our study sample where older adults who were nonfrail and older adults who were frail had significantly different total sleep durations (*P*=.01). Older adults who were nonfrail had a mean total sleep duration of 6.48 hours (close to 7 hours), while their frail counterparts slept for 8.16 hours. These findings demonstrate the additional information wearable devices provide over conventional sleep quality assessments.

In this study, we built logistic regression models using a sequential stepwise feature selection method. Feature selection in general can help improve predictive performance [53]. It minimizes the number of features needed in a model, which was critical given the small sample size of this study. While manual feature selection based on expert knowledge could have been a feasible alternative, our goal was to maximize frailty prediction performance in our data set by utilizing an empirical feature selection method. The analysis of multiple logistic regression models showed that wearable device data were a superior source of information for predicting frailty than sociodemographic information and patient assessments. However, the highest AUROC of 0.90 was achieved with the model that used wearable device data, sociodemographic, and patient assessment information. Previously, a similar study [16] that used a neck-worn wearable device to obtain step count and physical activity-related variables achieved an AUROC of 0.88 in discriminating the prefrail group from the frail and nonfrail groups. Another study [48] used 2 research-grade wearable devices concurrently and achieved an AUROC of 0.86 in discriminating 3 frailty states using stride length. Both studies were limited due to their short 48-hour observational period and being conducted in a laboratory setting. Our study demonstrated that unsupervised monitoring of frailty at home using a wearable device is possible. Our results corroborate that wearable technology should complement, rather than replace, the existing practice [54].

Many mobile health and telehealth apps have been successful at delivering health care while improving efficiency [55]. A study [56] that examined telehealth for frail older adults found the most cost-effective telehealth program used automated monitoring of vital signs to reduce health service use and facilitate remote follow-up. Wearable devices are becoming increasingly affordable and are capable of offering a similar use case as telehealth apps with their automated monitoring of physical activities, sleep, and heart rate. The range of information collected from wearable devices are also increasing with the advancement of sensor technology such as electrocardiogram, blood glucose level, oxygen saturation level, and electrodermal activity. When coupled with well-calibrated algorithms that enable early detection of health deteriorations such as frailty, cost savings can be further increased. The added value of wearable devices in assessing frailty for home care clients and community-dwelling older adults should be carefully evaluated for their feasibility in real-life settings. Each home or community health care system is unique, including but not limited to their target population, geographical area, and funding structure. Future research should consider these factors when evaluating the clinical value and cost savings of wearable devices.

Future research should confirm the predictive power of data derived from wearable devices and extend it beyond the home and community care sector. Our results indicated that wearable devices are a valid tool when an adequate analytical process is used. We recommend that future home care research studies leverage the potential of consumer-grade wearable devices to help identify vulnerable and frail groups who may benefit from additional home care services and increased access to health care.

### Limitations

Our study has several limitations. First, the small study sample prevented us from stratifying patients into nonfrail, prefrail, and frail groups. A third frailty state could have helped us demonstrate gradient measures of wearable data. The small sample size also limited the number of variables that could be used in developing multiple logistic regression models. The 3 logistic regression models were each fitted with 2 to 4 features. They exceeded the common rule of 1-in-10 and which may have increased the risk of overfitting [57]. The small sample size precluded partitioning our data into training and test sets. As a result, the reported predictive performance overestimated the performance that would be found on a different sample of older adults. A further caution should be taken when interpreting the results of the Hosmer-Lemeshow test due to the small sample size.

Our research used an 8-day observation period. While this was longer than the observation periods of most other studies using wearable devices, an even longer observational period may be required to reveal new patterns that are not observable within 8 days such as weekdays versus weekends and seasonal differences. Lastly, the validation studies that examined the Mi Band [32,33] were conducted in younger participants, limiting their generalizability to older adults of this study.

### Conclusions

In this study, we proved the concept of using a wrist-worn consumer-grade wearable device to assess frailty among older home care clients. Data collected from the wearable device, such as total sleep time and deep sleep time, were associated with frailty. The frailty prediction model based on variables selected from wearable devices, sociodemographic variable, and patient assessment variables achieved the highest AUROC of 0.90, compared to the AUROC of the other predictive models that either used only sociodemographic and assessment variables or only wearable device–derived variables.

## Appendix

Multimedia Appendix 1 The results of Shapiro-Wilk normality tests for all continuous variables.

Multimedia Appendix 2 T test statistics and chi-square test statistics for comparisons between the frail and nonfrail participants with respect to baseline sociodemographic and patient characteristics (n=37).

Multimedia Appendix 3 Boxplots of the wearable device data comparing frail and nonfrail participants.

## Abbreviations

AUROC: area under the receiver operating characteristics curve
CCI: Charlson comorbidity index
