# Supplementary material for: Consumer-Grade Wearable Device for Predicting Frailty in Canadian Home Care Service Clients: Prospective Observational Proof-of-Concept Study
Source: J Med Internet Res. 2020 Sep 3;22(9):e19732. doi: 10.2196/19732 (PMC7499164; doi:10.2196/19732)
Supplement: Multimedia Appendix 1 [file jmir_v22i9e19732_app1.docx]

Multimedia Appendix 1. The results of Shapiro-Wilk normality tests for all continuous variables

|  | W statistic value (p-value) | |  |
| --- | --- | --- | --- |
|  | Non-frail | Frail | Selected statistical test |
| Age | 0.94 (0.17) | 0.84 (0.02*) | Mann-Whitney U |
| BMI^a^ | 0.98 (0.90) | 0.92 (0.24) | t-test |
| ADL^b^ | 0.77 (<0.01*) | 0.85 (0.03*) | Mann-Whitney U |
| CCI^c^ | 0.88 (0.01*) | 0.93 (0.30) | Mann-Whitney U |
| Home care utilization | 0.88 (<0.01*) | 0.79 (<0.01*) | Mann-Whitney U |
| Worn time, hours per day | 0.89 (0.01*) | 0.92 (0.26) | Mann-Whitney U |
| Daily step count, n | 0.90 (0.02*) | 0.87 (0.06) | Mann-Whitney U |
| Deep sleep time, min | 0.84 (<0.01*) | 0.94 (0.43) | Mann-Whitney U |
| Light sleep time, min | 0.97 (0.78) | 0.90 (0.16) | t-test |
| Total sleep time, min | 0.95 (0.29) | 0.77 (<0.01*) | Mann-Whitney U |
| Awake time, min | 0.86 (<0.01*) | 0.87 (0.06) | Mann-Whitney U |
| Sleep quality, % | 0.69 (<0.01*) | 0.81 (0.01*) | Mann-Whitney U |
| Heart rate, bpm^d^ | 0.97 (0.65) | 0.95 (0.53) | t-test |
| Heart rate SD^e^, bpm | 0.96 (0.49) | 0.91 (0.16) | t-test |

^*^ p<0.05

^a^ body mass index

^b^ activities of daily living

^c^ Charlson comorbidity index

^d^ beats per minute

^e^ standard deviation
