# Supplementary material for: Consumer-Grade Wearable Device for Predicting Frailty in Canadian Home Care Service Clients: Prospective Observational Proof-of-Concept Study
Source: J Med Internet Res. 2020 Sep 3;22(9):e19732. doi: 10.2196/19732 (PMC7499164; doi:10.2196/19732)
Supplement: Multimedia Appendix 2 [file jmir_v22i9e19732_app2.docx]

Multimedia Appendix 2. t-statistics and chi-square statistics for comparisons between the frail and non-frail participants with respect to baseline sociodemographic and patient characteristics (n=37)

|  | Statistic value (df) | p-value | Performed statistical analysis |
| --- | --- | --- | --- |
| Age, years | 1369.00 | <0.01* | Mann-Whitney U |
| Sex | <0.01 (1) | 1.00 | Chi-square |
| BMI, kg/m^2 a^ | 0.73 (20.70) | 0.44 | t-test |
| ADL score ^b^ | 180.00 | 0.43 | Mann-Whitney U |
| CCI score ^c^ | 106.50 | 0.11 | Mann-Whitney U |
| Marital status | 3.76 (3) | 0.29 | Chi-square |
| Education | 2.45 (2) | 0.12 | Chi-square |
| Income | 7.34 (2) | 0.03* | Chi-square |
| Income - Prefer not to answer, n=10 | 5.36 (1) | 0.06 | Post-hoc |
| Low income, n=17 | 1.04 (1) | 0.93 | Post-hoc |
| Mid to high income, n=10 | 0.62 (1) | 1.00 | Post-hoc |
| Ethnicity | 0.13 (1) | 0.71 | Chi-square |
| Homecare Utilization |  |  |  |
| Personal support service, hours per week | 77.50 | 0.01* | Mann-Whitney U |

*p<0.05

^a^ body mass index

^b^ activities of daily living

^c^ Charlson comorbidity index
