# Supplementary material for: Consumer-Grade Wearable Device for Predicting Frailty in Canadian Home Care Service Clients: Prospective Observational Proof-of-Concept Study
Source: J Med Internet Res. 2020 Sep 3;22(9):e19732. doi: 10.2196/19732 (PMC7499164; doi:10.2196/19732)

Multimedia Appendix 3. Boxplots of the wearable device data comparing frail and non-frail participants


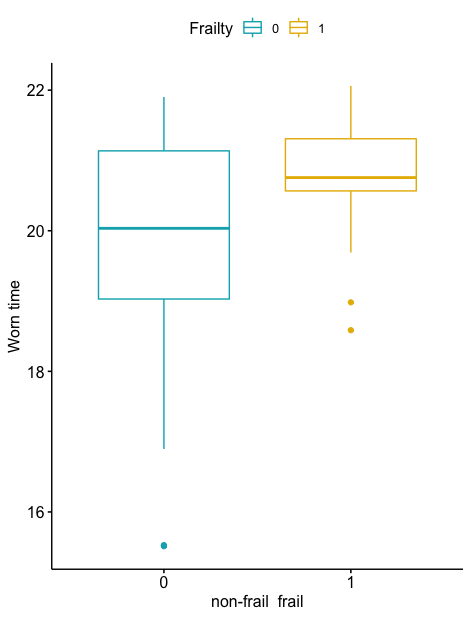

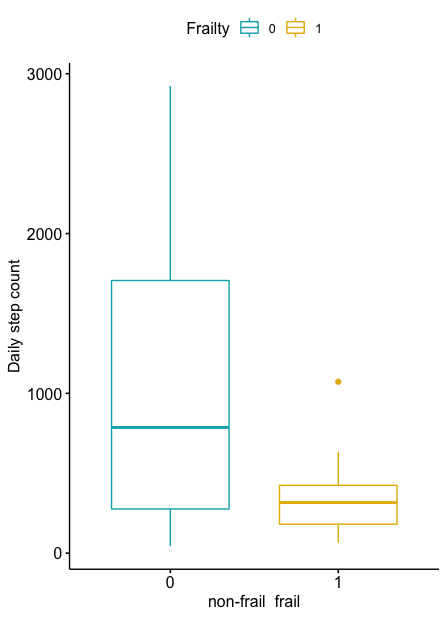

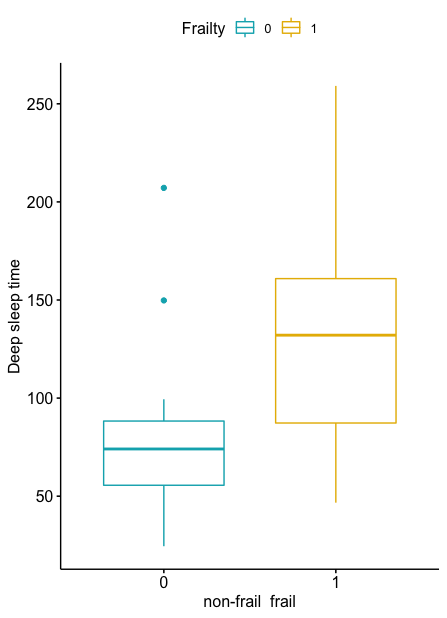

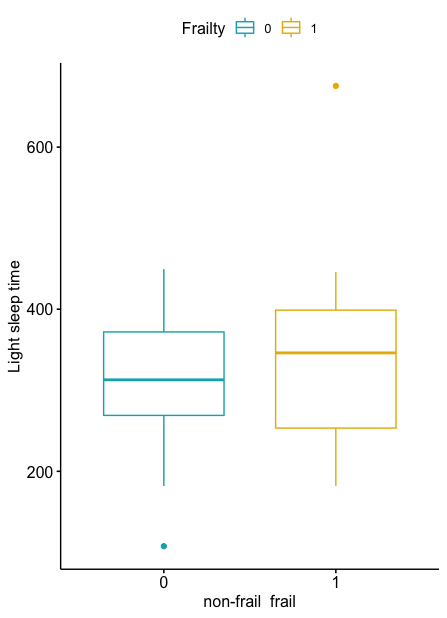

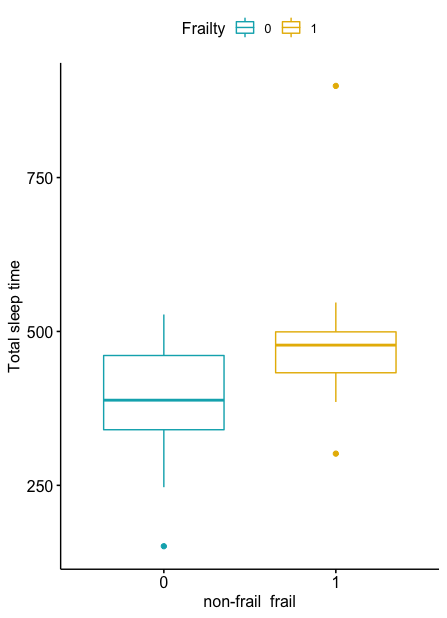

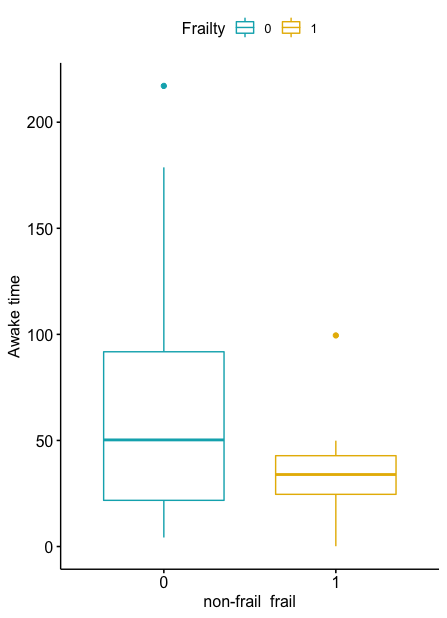

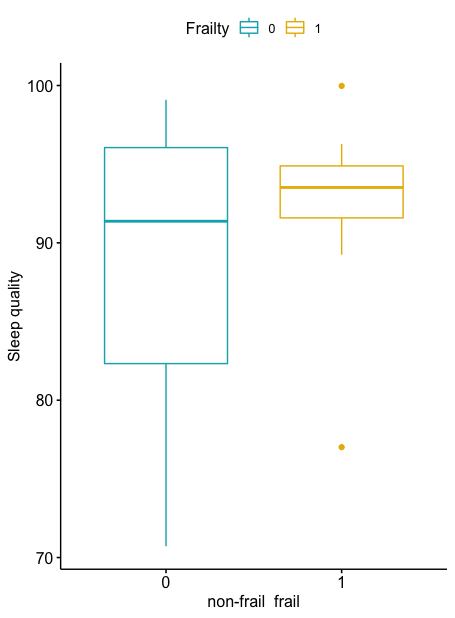

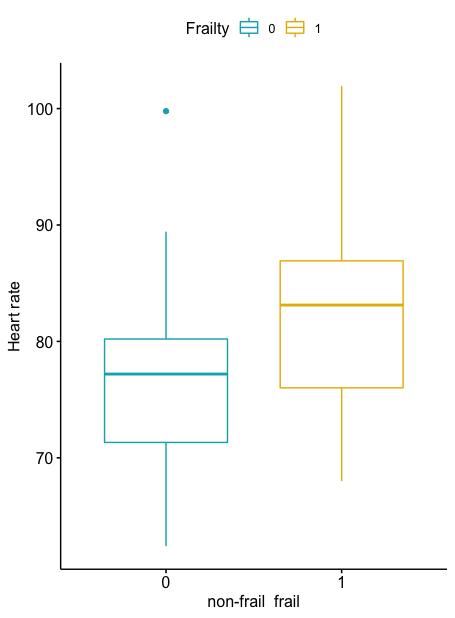

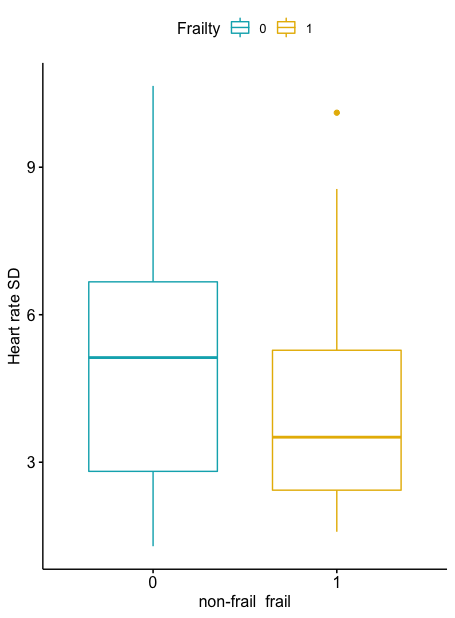

Supplement: Multimedia Appendix 3 [file jmir_v22i9e19732_app3.docx]
